# A tree frog (*Boana pugnax*) dataset of skin transcriptome for the identification of biomolecules with potential antimicrobial activities

**DOI:** 10.1016/j.dib.2020.106084

**Published:** 2020-07-25

**Authors:** Yamil Liscano Martinez, Claudia Marcela Arenas Gómez, Jeramiah Smith, Jean Paul Delgado

**Affiliations:** a Universidad de Antioquia, Sede de Investigacio´n Universitaria, Torre 2, laboratorio 432. Calle 62 No. 52 - 59., Carrera 53 # 61 - 30, Medellín, Antioquia, Colombia; b Department of Biology, University of Kentucky, Lexington, KY, USA

**Keywords:** *Boana pugnax*, Transcriptomics, Skin, Antimicrobial

## Abstract

Increases in the prevalence of multiply resistant microbes have necessitated the search for new molecules with antimicrobial properties. One noteworthy avenue in this search is inspired by the presence of native antimicrobial peptides in the skin of amphibians. Having the second highest diversity of frogs worldwide, Colombian anurans represent an extensive natural reservoir that could be tapped in this search. Among this diversity, species such as *Boana pugnax* (the Chirique-Flusse Treefrog) are particularly notable, in that they thrive in a diversity of marginal habitats, utilize both aquatic and arboreal habitats, and are members of one of few genera that are known to mount a robust immunological response against the fungus *Batrachochytrium dendrobatidis*, which has decimated the population of frogs worldwide. To search for molecules with potential antimicrobial activity, we have assembled and annotated a reference transcriptome from the skin of four wild captured *B. pugnax* from Antioquia, Colombia. Analysis of potential antimicrobial and immunological components was performed using ontology analyses, we identified several antimicrobial chemokines with particularly strong potential for exhibiting broadscale antimicrobial activities, as well as several genes related to rapid alteration of transcriptional (KRAB zinc finger protein) and phosphorylation (MAPK) responses to exogenous stressors. We also found eight families of transmembrane transport proteins, including sodium, potassium and voltage-dependent calcium channels, which will be invaluable in future studies aimed at more precisely defining the diversity and function of cationic antimicrobial peptides with alpha-helical structures. These data highlight the utility of frogs such as *Boana pugnax* in the search of new antimicrobial molecules. Moreover, the molecular datasets presented here allow us to expand our knowledge of this species and illustrate the importance of preserving the vast potential of Colombian biodiversity for the identification of useful biomolecules.

**Specifications Table**

**Table:** 

**Subject**	Animal Science and Zoology
**Specific subject area**	Wild caught adult frogs of the specie *Boana pugnax* from the Andes region of Antioquia, Colombia.
**Type of data**	RNA Sequencing Data
**How data were acquired**	Paired-End sequenced (2 × 100 bp) using an Illumina Hiseq-4000
**Data format**	Raw Sequencing reads, assembled contigs and preliminary annotation.
**Parameters for data collection**	Adult animals were used to surgically collect skin tissue (dorsal and ventral skin)
**Description of data collection**	Tissues were collected from animals following euthanasia via immersion in 200 mg/kg buffered tricaine. All samples (dorsal and ventral) were stored at —20 °C in RNAshield^Ⓡ^ reagent by one week until total RNA was extracted individually from each tissue.
	Paired-End sequenced (2 × 100 bp) using an Illumina Hiseq-4000.
**Data source location**	Institution: Universidad de Antioquia City/Town/Region: Antioquia Country: Colombia
	Latitude and longitude (and GPS coordinates) for collected samples/data: 6°15′49.6″N
	75○ 25′18.3″W
**Data accessibility**	Repository name: Sequence Read Archive
	Data identification number: SRP151854
	Direct URL to data: https://www.ncbi.nlm.nih.gov/sra/?term=boana±pugnax
	Repository name: Transcriptome Shotgun Assembly
	Data identification number: GINY010000001 Direct URL to data:
	https://www.ncbi.nlm.nih.gov/Traces/wgs/GHME01?val=GINY01_accs

**Value of the data  **

• We describe a *de novo* reference transcriptome for a treefrog, *Boana pugnax* (Anura: Hylidae).
• There are few transcriptomic data on Colombian frogs and frogs from the family Hylidae. Such data will aid in understanding the evolution of mechanism that contribute to protection against pathogenic microorganisms and bioprospecting of peptides with antimicrobial activity.
• This study will provide useful comparative data for similar studies in other frogs and in the search for antimicrobial agents in *Boana pugnax*.

## 1 Data description

### 1.1 RNA sequencing and transcriptome assembly

**Fig. 1:**
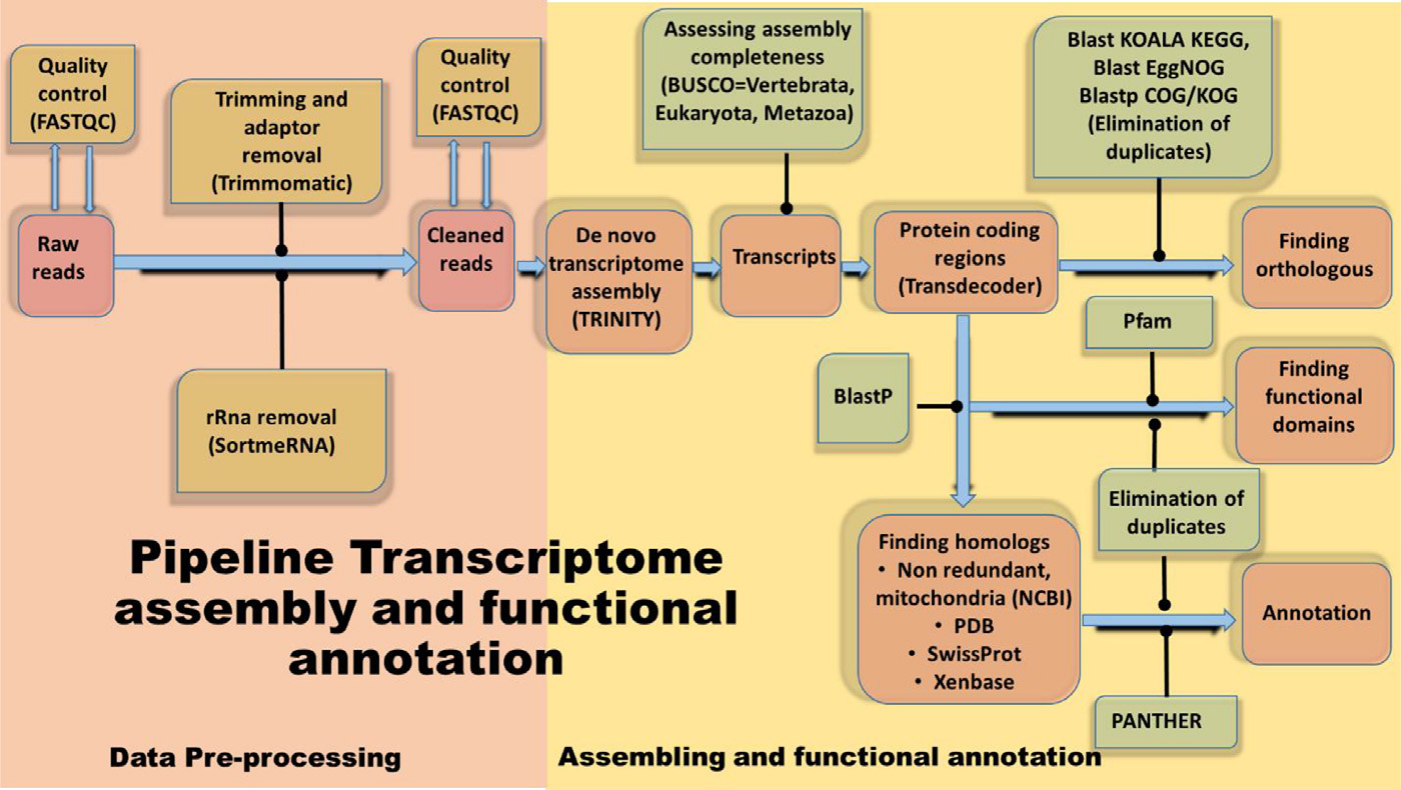
Workflow for data processing, assembly and annotation of *B.pugnax* mRNAs.

**Fig. 2:**
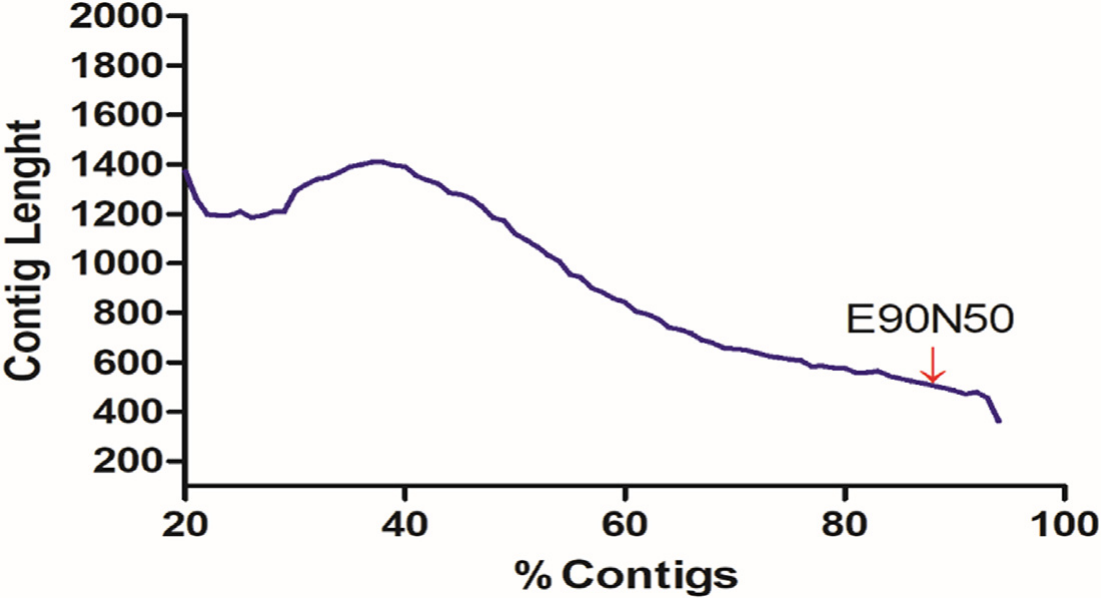
E90N50 from *B.pugnax* transcriptome.

Sequencing yielded a total of 809,394,848 raw reads. Prior to assembly (Fig. 1), adapter sequences and ribosomal RNAs were filtered from the population of raw reads leaving a total of 573,994,007 clean reads (Specifications table). Assembly of these reads yielded a collection of 1,136,865 transcripts with an average GC content of 43.0%, which is similar to that of other frogs such as *Rana temporaria* 44% [1] and *Oreobates cruralis* 45% [2]. The alignment-free abundance estimation methods Kallisto was used to filter the transcripts based on expression values [4]. A total of 1,008,042 transcript were retained after filtering the most highly expressed isoform per gene and from those 252,491 were transcriptionally supported isoforms (> 1 TPM, Transcripts per Million). The quality of the assembly was evaluated using BUSCO [5] and by calculating the E90N50 (N50 for the top 90% expressed transcripts), resulting in a value of 600 bp (Fig. 2)

### 1.2 Annotation

**Fig. 3:**
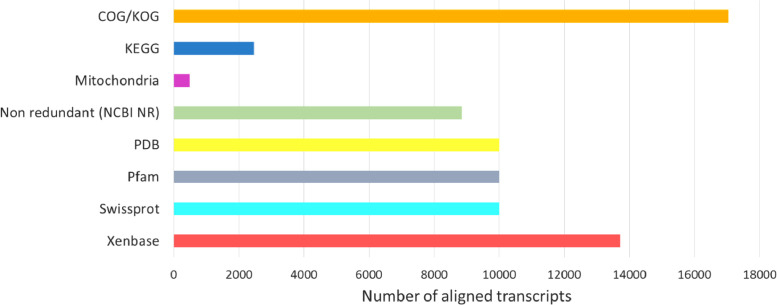
Number of transcripts aligned to various databases. There were eight databases with 104,603,835 proteins used to align the transcripts. The largest number of them was aligned to the COG/KOG database, followed by the Xenopus database that includes the two species, *X. laevis* and *X.tropicalis*. Other databases with an alignment of 10,000 transcripts were Swissprot, PDB and Pfam. Among the databases with the lowest number of transcripts are KEGG and the mitochondria of NCBI.

Using Transdecoder, we identified 112,473 candidate open reading frames. Putative homologs were identified for 72,018 of these by alignment to multiple databases. The largest number of predicted protein alignments were found in searches against the COG / KOG database with 17,047, followed by Xenbase with 13,174, Pfam, PDB, and Swissprot with 10,000, NCBI non-redundant 8850, KEGG with 2461 and Mitochondria with 486 as shown in Fig. 3

**Fig. 4:**
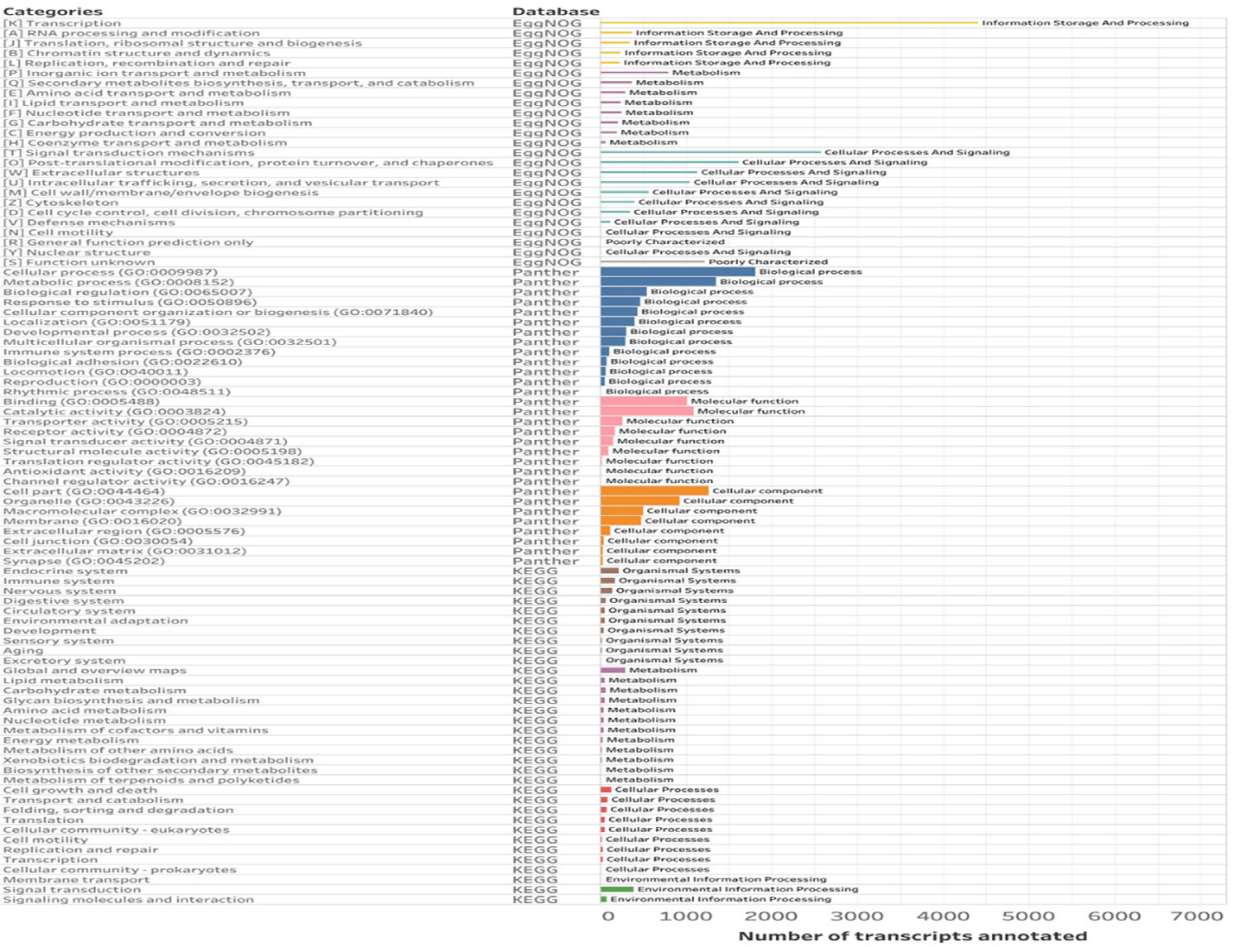
Distribution of terms Gene ontology (GO) for the transcriptome of *B. pugnax* using PANTHER3.1. EggNOG and KEGG database.

Building on homology-based annotations, functional annotations were carried out to more fully characterize functions represented within the skin and to specifically identify genes and pathways associated with innate defense. For this we used PANTHER (information from 4242 genes), EggNOG (information from 17,047 genes) and KEGG (information from 2461 genes), (Fig. 4).

**Fig. 5:**
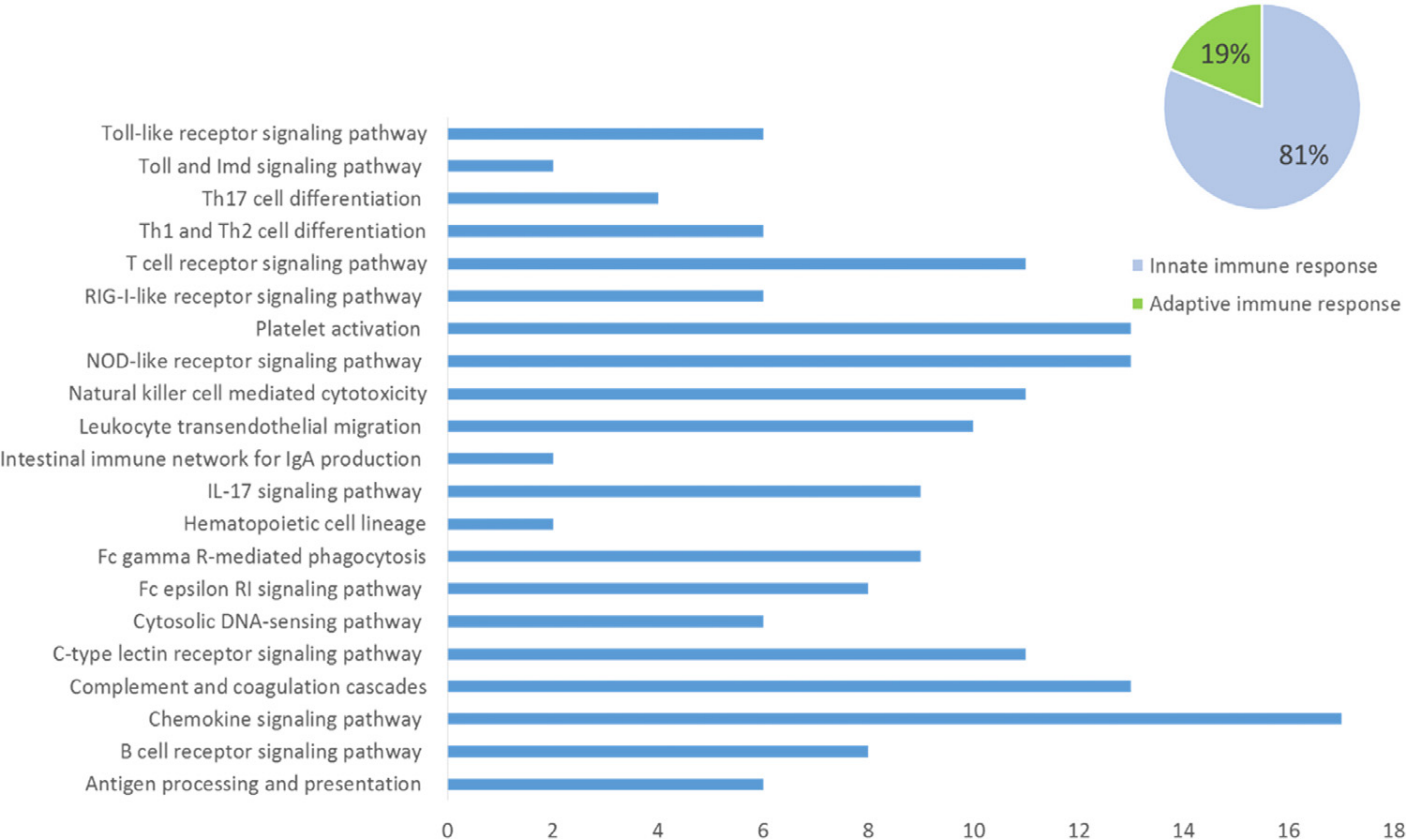
Putative genes related to pathways and its association to immune response. The pathways related tothe immune system to which these genes were associated mainly to chemokine signaling pathway. These components areassociated with 81% of the innate immune system and 19% of the adaptive system.

In the analyzes of proteins involved in the immune system pathways using PANTHER pathways, we found 21 proteins, of these 17 (81%) are related to the innate immune response and four (19%) to the adaptive immune response (Fig. 5). Pathways related to innate immunity included several genes related to inflammation mediated by cytokines and chemokines and the MAP kinase signaling pathway, which integrates transduction of stress responses to mediate transcription. Among the proteins associated with chemokine signaling were several receptors of chemokines, including: C-X-C chemokine receptor type 2 (CXCR2, receptor of CXCL1 and CXCL2), C-X-C chemokine receptor type 3 (CXCR3, receptor of CXCL9 and CXCL10), C—C chemokine receptor type 7 (CCR7), C—C chemokine receptor type 8 (CCR8), and C—C chemokine receptor type 9 (CCR9), and the GTPase KRas. The latter encodes for the RAS protein that is part of a signaling pathway known as the RAS / MAPK pathway (Furukawa, 2015). The NOD-like receptor signaling pathway, complement, and coagulation cascades and platelet activation pathways were also highly represented. These pathways share a common feature in that they all contribute to the first line of defense against pathogens and other invaders, the innate response [5], [6].

### 1.3 Protein domain identification using Pfam

To supplement functional information gathered from homology-based studies, we also performed analyses to identify enriched domains within the skin transcriptome. In general, the most frequent domains in the transcriptome were those involved in transcriptional regulation, signal transduction pathways of the immune system and cellular proliferation (Table 1). The 10 most frequent domains were Zinc fingers (PF00096: 189 transcripts), followed by the Endonuclease domains (PF03372: 67 transcripts) presumably from transposons, transposable elements (PF02994: 49 transcripts), reprolysin (PF01421: 26 transcripts), trypsin (PF00089: 23 transcripts), aldo-keto reductase (PF00248: 21 transcripts), calcium-binding EGF (PF07645: 18 transcripts), cytochrome P450 (PF00067: 16 transcripts), protein kinase (PF00069: 16 transcripts) and ATP-binding cassette transporter (PF12698: 15 transcripts). These domains are related to cellular signaling processes (Zinc finger, calcium-binding EGF), post-translational modifications, protein turnover (reprolysin, trypsin), biosynthesis of secondary metabolites (C.p450, ATP binding cassette transporter), production and conversion of energy (aldo-keto-reductase).

**Table 1:** Top ten Pfam domains within the *B. pugnax* transcriptome.

Pfam domain	N-hits	Pfam ID
Zinc finger	189	PF00096
Endonuclease/Exonuclease/phosphatase family	67	PF03372
L1 transposable element RBD-like domain	49	PF02994
Reprolysin (M12B) family zinc metalloprotease	26	PF01421
Trypsin	23	PF00089
Aldo-keto reductase	21	PF00248
Calcium-binding EGF	18	PF07645
Cytochrome P450	16	PF00067
Protein kinase	16	PF00069
ATP-binding cassette transporter	15	PF12698

### 1.4 Differential expression between dorsal and ventral skin

**Fig. 6:**
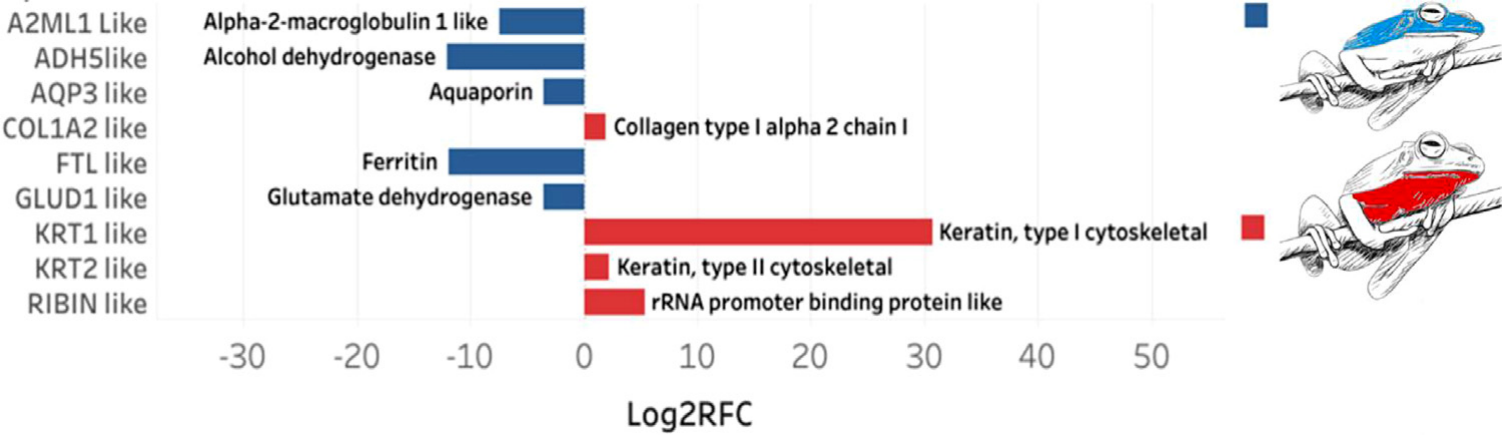
Differentially expressed genes between the dorsal and ventral skins of *B.pugnax.*

Fig. 6 shows the differential expression of proteins in the dorsal and ventral region of the skin of *B. pugnax*. The presence of Alcohol dehydrogenase, Aquaporin, Ferritin, Glutamate dehydrogenase and Alpha 2-macroglobulin is evident in the dorsal region. Keratin Type I, Keratin Type II, Collagen Type I and rRNA promoter binding protein are expressed more in the ventral region.

### 1.5 Transmembrane transport complexes with alpha helical structure

**Fig. 7:**
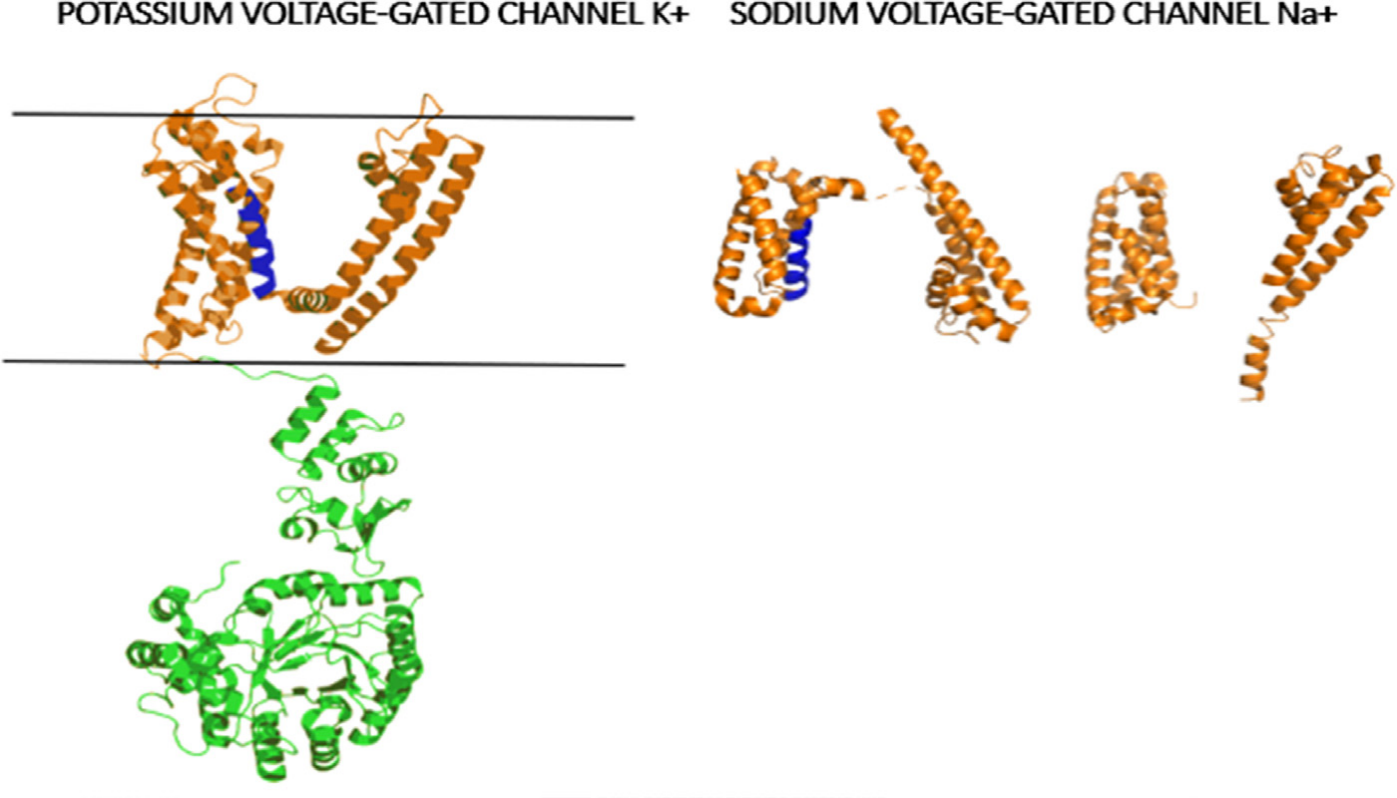
Structure of voltage-gated channels of sodium (4VGN) and potassium (2R9R), forming alpha helical secondary structure. The transmembrane regions are highlighted in orange and regions highlighted in green are predicted to reside in the cytoplasm . Regions highlighted in blue are considered candidates for new antimicrobial peptides.

We found 32 families of proteins and 8 classes of proteins within the search for transmembrane transport complexes in the transcriptome of *B. pugnax* (Table 2). Among these are the transmembrane receptor regulatory/adaptor protein (PC00226), the voltage-gated calcium channel (PC00240), the voltage-gated potassium channel (PC00242), the voltage-gated sodium channel (PC00243), the ion channel (PC00133), the ligand-gated ion channel (PC00141), the GABA receptor (PC00023) and the acetylcholine receptor (PC00037). All of these gens ae predicted to possess transmembrane alpha helical secondary structures (Fig. 7).

**Table 2:** Protein families of membrane receptors possessing alpha helical structures

Protein family	Alpha helix
GLUTAMATE RECEPTOR IONOTROPIC, KAINATE 1 (PTHR18966:SF36)	Yes
GLUTAMATE RECEPTOR IONOTROPIC, NMDA 2C (PTHR18966:SF361)	Yes
VOLTAGE-DEPENDENT CALCIUM CHANNEL SUBUNIT ALPHA-2/DELTA-2 (PTHR10166:SF7)	Yes
DISKS LARGE HOMOLOG 1 (PTHR23119:SF5)	Yes
LEUCINE-RICH REPEAT-CONTAINING PROTEIN 7 (PTHR23119:SF48)	Yes
POTASSIUM VOLTAGE-GATED CHANNEL SUBFAMILY G MEMBER 3 (PTHR11537:SF91)	Yes
VOLTAGE-DEPENDENT L-TYPE CALCIUM CHANNEL SUBUNIT BETA-1 (PTHR11824:SF17)	Yes
CALCIUM-ACTIVATED POTASSIUM CHANNEL SUBUNIT BETA-4 (PTHR10258:SF3)	Yes
POTASSIUM VOLTAGE-GATED CHANNEL SUBFAMILY KQT MEMBER 4 (PTHR11537:SF4)	Yes
VOLTAGE-DEPENDENT CALCIUM CHANNEL SUBUNIT ALPHA-2/DELTA-4 (PTHR10166:SF59)	Yes
POTASSIUM VOLTAGE-GATED CHANNEL SUBFAMILY A MEMBER 2 (PTHR11537:SF23)	Yes
POTASSIUM VOLTAGE-GATED CHANNEL SUBFAMILY A MEMBER 4 (PTHR11537:SF45)	Yes
GLUTAMATE RECEPTOR 1 (PTHR18966:SF157)	Yes
DISKS LARGE HOMOLOG 3 (PTHR23119:SF7)	Yes
SODIUM CHANNEL PROTEIN TYPE 5 SUBUNIT ALPHA (PTHR10037:SF206)	Yes
SODIUM CHANNEL SUBUNIT BETA-3 (PTHR10546:SF1)	Yes
GLUTAMATE RECEPTOR IONOTROPIC, KAINATE 2 (PTHR18966:SF38)	Yes
POTASSIUM VOLTAGE-GATED CHANNEL SUBFAMILY D MEMBER 3 (PTHR11537:SF182)	Yes
SHORT TRANSIENT RECEPTOR POTENTIAL CHANNEL 6 (PTHR10117:SF7)	Yes
GLUTAMATE RECEPTOR IONOTROPIC, NMDA 2B (PTHR18966:SF382)	Yes
CALCIUM UNIPORTER PROTEIN, MITOCHONDRIAL (PTHR13462:SF16)	Yes
VOLTAGE-DEPENDENT L-TYPE CALCIUM CHANNEL SUBUNIT BETA-3 (PTHR11824:SF8)	Yes
POTASSIUM VOLTAGE-GATED CHANNEL SUBFAMILY KQT MEMBER 5 (PTHR11537:SF128)	Yes
VOLTAGE-DEPENDENT T-TYPE CALCIUM CHANNEL SUBUNIT ALPHA-1H (PTHR10037:SF192)	Yes
RYANODINE RECEPTOR 3 (PTHR13715:SF16)	Yes
VOLTAGE-DEPENDENT L-TYPE CALCIUM CHANNEL SUBUNIT BETA-2 (PTHR11824:SF9)	Yes
SODIUM CHANNEL SUBUNIT BETA-1 (PTHR10546:SF2)	Yes
ESSENTIAL MCU REGULATOR, MITOCHONDRIAL (PTHR33904:SF1)	Yes
POTASSIUM VOLTAGE-GATED CHANNEL SUBFAMILY A MEMBER 6 (PTHR11537:SF104)	Yes
NEURONAL ACETYLCHOLINE RECEPTOR SUBUNIT BETA-3 (PTHR18945:SF75)	Yes

## 2 Experimental design, materials, and methods

### 2.1 Ethics approval

The Ethics Committee for Animal Experimentation of the University of Antioquia approved this project, under endorsement 569. Specimen collection and access to genetic resources was approved by the Ministry of Environment on September 24, 2015 for non-commercial research number 119. Collections are deposited under record 080 at the Museum of Herpetology University of Antioquia (MHUA) within the biological collections of the Alexander von Humboldt Institute.

### 2.2 Sample collection

Four individuals of the species *Boana pugnax* (*B. pugnax)* were collected in Vegas de la Clara (University of Antioquia property)latitude 66.815.224 longitude -752.194.104, Antioquia, Colombia, on June 13, 2016 at an average temperature of 25 °C. The individuals had an average weight of 9.11 g (10 g, 6 g, 9 g, 11.47 g) and an average length of 6.25 cm (6 cm, 5.5 cm, 6.5 cm, 7 cm). Animals were transported to the laboratory in sterile plastic bags at 25 °C.

### 2.3 Sample preparation for RNA-seq

Frogs were anestietized and euthanized by intercardial injection of 200 mg/kg buffered tricaine, after which the dorsal and ventral skin was removed under sterile conditions and deposited in 2 mL vials with 1.5 mL RNAshield (ZYMO REASEARCH) to avoid RNA degradation. We designated each frog as frog 1, 2, 3 or 4, with 2 samples collected per individual, totaling 8 samples. Pooling biological replicate RNA samples, such as those derived from a number of experimentally similar animals, may retain biological information, while reducing the cost of sequencing [6], [7]. We therefore mixed the tissues of frog 1 with 2 and 3 with 4, prior to RNA extraction for a total of 4 samples (frog 1–2 ventral, frog 1–2 dorsal, frog 3–4 dorsal and frog 3–4 ventral).

We used a TissueLyser II^Ⓡ^ Qiagen to homogenize the tissues at 30 oscillations/second for 10 min. After tissue maceration, we add 1 mL TRIZOL^Ⓡ^ (Life Technologies) to each sample for the extraction of RNA. Total RNA was resuspended in 300 μL of Shield^Ⓡ^ Zymo RNA stabilization buffer and shipped to Macrogen for library preparation and sequencing. The integrity of the RNA (RIN) was verified using an Agilent 2100 Bioanalyser (Agilent Technologies) and all samples yielded RIN scores >8, which was considered of sufficiently high quality for library preparation [8]. The construction of 4 barcoded the TruSeq (Ilumina) cDNA libraries was carried out according to manufacturer protocols and the resulting libraries were paired-end sequenced (2 × 100 bp) using an Illumina HiSeq 4000 with a depth of 40 million reads per sample.

### 2.4 Bioinformatic analysis

#### 2.4.1 Read filtering

Verification of read quality was carried out using the FASTQC software [8] https://www.bioinformatics.babraham.ac.uk/projects/fastqc/). Reads were subsequently trimmed to remove sequencing adapters and ribosomal RNA in order to improve the accuracy of transcriptome assembly (Fig. 1). Prior to assembly, reads were filtered using SORTMERNA-v2.1 [9] in order to remove reads derived from ribosomal RNAs. This filtering step used the databases SILVA 16S bacteria, SILVA 16S archaea, SILVA 18S eukarya, SILVA 23S bacteria, SILVA 187 23s archaea, SILVA 28S eukarya, Rfam 5S archaea / bacteria, Rfam 5.8S eukarya. We then removed sequencing and PCR adapters with TRIMMOMATIC-v0.32 [10]. Finally, FASTQC was used to review post filtered read quality before proceeding with de novo assembly of the transcriptome.

#### 2.4.2 Transcriptome assembly

Filtered reads were assembled using Trinity v2.9.0 [11]. Assembly completeness was assessed using BUSCO-v1 to identify conserved orthologs in the Vertebrata, Eukaryota and Metazoa databases [5]. Following the pipeline reported by Trinity (https://github.com/trinityrnaseq/trinityrnaseq/wiki/Trinity-Transcript-Quantification) the alignment-free abundance estimation methods Kallisto was used to filter the transcripts based on expression values [3]. The transcriptome of *B. pugnax* was deposited in GenBank under SRA accession number: SRP151854 (BioProject PRJNA476387). Coding regions were predicted based on the identification of the longest ORF using TransDecoder-v3 [11].

#### 2.4.3 Functional annotation

Homology and domain-based annotations were performed in order to infer the function of predicted coding sequences [2]. To identify homologous sequences for functional annotation, transcripts were aligned to the SwissProt databases (ftp://ftp.ebi.ac.uk/pub/databases/uniprot/), NCBI non-redundant (ftp://ftp.ncbi.nih.gov/blast/db/FASTA/nr.gz) mitochondria (HmtDB: http://www.hmtdb.uniba.it/dbfunctions), protein data bank (PDB) and the Xenbase proteome (http://www.xenbase.org/, RRID:SCR_003280) using BLASTp with an e-value cutoff of <1.0e-5. The best hit for each *B. pugnax* transcript was used to extract functional annotation via PANTHER14.1 [12]. For inferring high-level functions we used three ontology databases: COG/KOG (ftp://ftp.ncbi.nih.gov/pub/COG/), EggNOG (Evolutionary Genealogy of Genes: Non-supervised Orthologous Groups) [13] and KEGG (Kyoto Encyclopedia of Genes and Genomes) [14]. We also identified functional domains by performing Blastp searches against the Pfam-A database [15].

#### 2.4.4 Transcript abundance and expression level analyses

Sequence reads generated from dorsal and ventral skin samples were aligned to the reference transcriptome using Bowtie2 [16], and RSEM (RNA-Seq by Expectation Maximization) was used to obtain estimates of transcript abundance for all transcripts [17]. Expression levels were calculated as transcripts per million (TPM). Differential expression of genes was evaluated statistically using EBseq. Transcripts were considered differentially expressed between samples (ventral vs dorsal) with FDR (False Discovery Rate) < 0.05. This list of differentially expressed genes was used for analyses of gene ontology enrichment using EggNOG, PantherDB and KEGG pathways as described above.

#### 2.4.5 Identification of transmembrane transport complexes with alpha helices

We used Panther to identify transmembrane transport proteins that will serve as a template to identify new antimicrobial peptide sequences. Once the proteins were obtained, we search their structure in the protein data bank (https://www.rcsb.org/) to select those with alpha helices in the transmembrane region.

## CRediT authorship contribution statement

**Yamil Liscano Martinez:** Investigation, Methodology, Data curation, Writing - original draft, Formal analysis. **Claudia Marcela Arenas Gómez:** Methodology, Writing - review & editing, Formal analysis. **Jeramiah Smith:** Methodology, Writing - review & editing. **Jean Paul Delgado:** Conceptualization, Project administration, Writing - review & editing.

## Declaration of Competing Interest

The authors declare that they have no known competing financial interests or personal relationships which have, or could be perceived to have, influenced the work reported in this article.

## Appendix Supplementary materials

Image, application 1
